# Characterization of the complete mitochondrial genome of *Ectropis grisescens* (Lepidoptera, Geometridae)

**DOI:** 10.1080/23802359.2021.1923423

**Published:** 2021-06-14

**Authors:** Xu-Hao Song, Ting-Bang Yang, Xiao-Qin Xu, Xiang-Hui Yan, Cai-Quan Zhou

**Affiliations:** a Key Laboratory of Southwest China Wildlife Resources Conservation (Ministry of Education), China West Normal University, Nanchong, Sichuan, China; b Institute of Ecology, China West Normal University, Nanchong, Sichuan, China

**Keywords:** *Ectropis grisescens*, mitochondrial genome, phylogenetic analysis, tea pest

## Abstract

*Ectropis grisescens* (Lepidoptera, Geometridae) is one of the main leaf-eating pests in tea plantations in China. In this study, the complete mitochondrial genome of this species was sequenced and assembled. The total length of the mitochondrial genome of *E*. *grisescens* was 15,794 bp (GenBank accession No. MW337302). The base composition was 41.26% for A, 39.49% for T, 7.92% for G, and 11.33% for C. The circular mitogenome contained 13 protein-coding genes, 2 ribosomal RNA genes, and 22 transfer RNA genes. Phylogenetic analysis performed using 13 protein-coding genes of 15 species of Geometridae and an out-group *Pieris melete* (Lepidoptera, Pieridae) showed that *E. grisescens* is closely related to species of *E*. *obliqua*, and this is consistent with the morphological identification.

Tea (*Camellia sinensis*) is an important cash crop that is widely cultivated in China, India, Ceylon, Indonesia and some African countries (Cranham 1966). *Ectropis grisescens* (Lepidoptera, Geometridae) is one of the main leaf-eating pests in tea plantations in China (Ge et al. 2016; Li et al. 2019). The occurrence of this tea pest occurs throughout the year. The yield of tea from plantations is seriously reduced by high numbers of this pest (Li et al. 2019). The morphological characteristics of *E*. *grisescens* are similar to those of *E. obliqua* (Lepidoptera, Geometridae), which is also one of the main leaf-eating pests in Chinese tea plantations (Zhang et al. 2014; Yang et al. 2017). Therefore, some tea growers and tea plant protection technicians have long regarded these two looper species as one species, collectively known as the tea geometrid. Tang et al. (2019) compared the morphological differences between these two looper species (including larvae and adults) through laboratory feeding observations and further verified them through DNA barcoding, accurately distinguishing *E*. *obliqua* and *E*. *grisescens*. Identification of these two looper species made it clear that *E*. *obliqua* is mainly distributed in tea areas of Zhejiang, Jiangsu and Anhui Provinces of China, whereas *E*. *grisescens* is widely distributed in all tea production areas of China (Li et al. 2019). Considering the negative impact of these two looper species in tea plantations, it is essential to identify their complete mitochondrial genomes and the related phylogenies (Liu et al. 2017). To date, the complete mitochondrial genome of *E*. *obliqua* has been deposited in GenBank (Wang et al. 2017). However, the complete mitochondrial genome of *E*. *grisescens* was still lacking. The mitochondrial genome of *E. grisescens* reported here will promote further understanding of the evolution, taxonomy, and population genetics of this main tea pest.

The specimens were collected from a tea plantation in Pujiang County (103.37 E; 30.19 N), Chengdu city, Sichuan Province, China, in October 2019, placed in plastic collection bottles (200 mL) with 100% ethanol, and stored at −20 °C. The collected loopers were identified as *E. grisescens* based on morphological characteristics (Tang et al. 2019). All specimens were deposited at Institute of Ecology, China West Normal University, Nanchong, Sichuan, China (https://ioe.cwnu.edu.cn/, contact person and email: Ting-bang Yang, tingbang_yang@aliyun.com) under the voucher number SC-2019-ILG-01. Genomic DNA of *E. grisescens* was extracted using the 2 × CTAB method (Vallet et al. 2008), and sequenced by Shanghai Personal Biotechnology Co., Ltd. (Shanghai, China). Sequencing was performed by the Illumina NovaSeq platform with 2 × 150 bp paired-end reads (Illumina, San Diego, CA, USA). Total 15,681,406 raw reads were obtained. AdapterRemoval v2 software was used to filter the data and generate high-quality sequences (Schubert et al. 2016). Total 11,838,930 high-quality reads were obtained. High-quality reads were then used to produce a denovo assembly using A5-miseq v20150522 (Coil et al. 2014) and SPAdes v3.9.0 software (Bankevich et al. 2012) with different k-mer sizes (21, 55, 85, 115). Gaps among contigs were filled using MUMmer v3.1 software (Kurtz et al. 2004). Protein-coding genes (PCGs), ribosomal RNA (rRNA), and transfer RNA (tRNA) genes were predicted by using MITOS tools (Bernt et al. 2013).

The total length of the mitochondrial genome of *E. grisescens* was 15,794 bp (GenBank accession No. MW337302). The circular mitogenome contained 13 PCGs, 2 rRNA genes, and 22 tRNA genes. The base composition of the genome was as follows: A (41.26%), T (39.49%), G (7.92%), and C (11.33%).

To validate the phylogenetic position of *E. grisescens*, 13 PCGs of 15 species of Geometridae and an out-group *Pieris melete* (Lepidoptera, Pieridae) were aligned together with *E. grisescens* to construct the phylogenetic trees using Bayesian inference phylogenetic methods as described by Song et al. (2015). The gaps and ambiguous sites adjacent to gaps and stop codons were deleted, leaving 11,022 bp for phylogenetic analyses. Multiple alignments of the concatenated sequences from *E. grisescens* and 16 other species were performed using ClustalX 2.1 software (Thompson et al. 1997) with the default settings. Bayesian phylogenetic analyses were performed using MrBayes 3.1.2 (Ronquist and Huelsenbeck, 2003). The TIMef substitution model was selected for Bayesian analyses using ModelTest v3.7 (Posada and Crandall 1998) under the Akaike Information Criterion (AIC) (Posada and Buckley 2004). Bayesian posterior probabilities were estimated using the Markov chain Monte Carlo (MCMC) sampling approach. The program was initiated with randomly generated trees and ran for ten million generations, sampling one tree per 1000 generations, and then the first 25% of these sampled trees were discarded as burn-in. Finally, a majority-rule consensus tree was generated from the remaining trees. As shown in the phylogenetic tree (Figure 1), *E. grisescens* and *E*. *obliqua* were clustered into a branch. This suggests that *E. grisescens* is closely related to species of *E*. *obliqua*, and this is consistent with the morphological identification.

**Figure 1.**
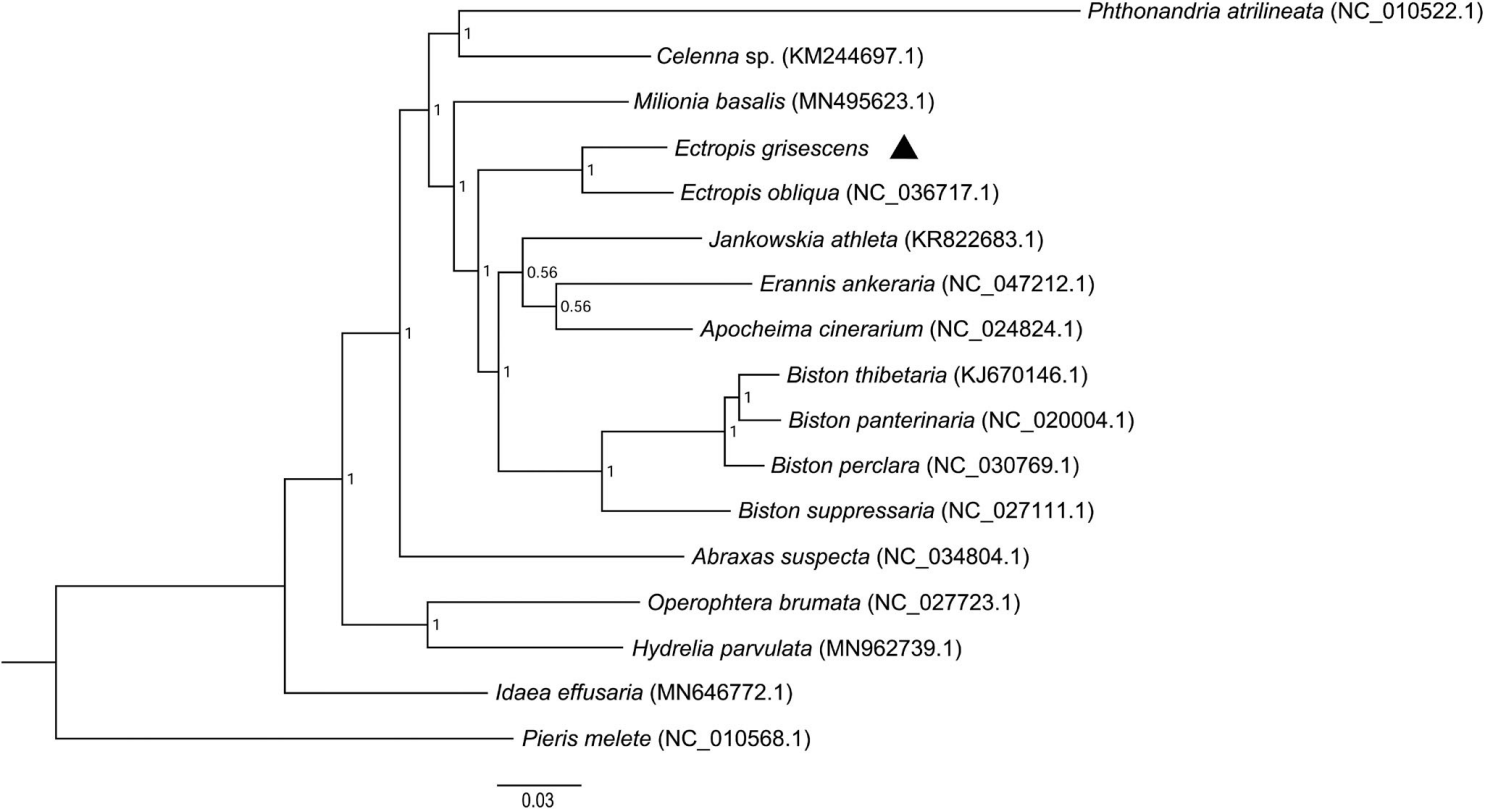
Molecular phylogeny of *Ectropis grisescens* and the related species in the family Geometridae based on 13 protein-coding genes. The phylogenetic tree was constructed by Bayesian inference phylogenetic method. The GenBank accession numbers are listed after the scientific names of the species. The position of *E. grisescens* is marked in triangle.

## Data Availability

The genome sequence data that support the findings of this study are openly available in GenBank of NCBI at https://www.ncbi.nlm.nih.gov/ under the accession no. MW337302. The associated BioProject, SRA, and Bio-Sample numbers are PRJNA717817, SRR14085641, and SAMN18515037 respectively.
